# Clinical Features and Analysis in Pituitary Stalk Interruption Syndrome

**DOI:** 10.1155/2024/2493083

**Published:** 2024-05-24

**Authors:** Qiuxuan Guo, Jing Zhao, Shuang Yu

**Affiliations:** ^1^Department of Endocrinology, Fuqing City Hospital Affiliated to Fujian Medical University, Fuqing 350300, Fujian, China; ^2^Department of Radiology, The First Affiliated Hospital of Sun Yat-sen University, Guangzhou 510080, Guangdong, China; ^3^Department of Endocrinology, The First Affiliated Hospital of Sun Yat-sen University, Guangzhou 510080, Guangdong, China

## Abstract

**Objective:**

Pituitary stalk interruption syndrome (PSIS) is characterized by the absence of pituitary stalk, pituitary hypoplasia, and ectopic posterior pituitary. Because the etiology and clinical cognition of PSIS remain elusive, we analyzed the clinical features of PSIS in Chinese patients.

**Methods:**

A retrospective analysis was conducted on the clinical presentation, laboratory data, imaging examination, and management of 24 PSIS inpatients from our center over 10 years.

**Results:**

Among the 24 PSIS patients, there were 22 males (91.7%) and 2 females (8.3%). Growth hormone deficiency was present in all 24 cases (100%), hypogonadism in 24 cases (100%), secondary adrenal insufficiency in 22 cases (91.2%), and hypothyroidism in 21 cases (87.5%). 20 cases (83.3%) of PSIS patients exhibited deficiencies in four anterior pituitary hormones, 3 cases (12.5%) exhibited deficiencies in three anterior pituitary hormones, and 1 case (4.2%) exhibited deficiencies in two anterior pituitary hormones, with none exhibiting deficiencies in posterior pituitary hormones. Among the 24 PSIS patients, 12 had a history of growth hormone therapy before admission, and 12 had no such history. Additionally, 19 cases (79.2%) with PSIS were complicated by dyslipidemia, 15 cases (62.5%) were complicated by nonalcoholic fatty liver disease, and 9 cases (37.5%) were complicated by hyperuricemia.

**Conclusions:**

PSIS often presents with growth retardation and hypogonadotropic hypogonadism, but in some cases, short stature is not exhibited. PSIS is prone to complications such as dyslipidemia, nonalcoholic fatty liver disease, and hyperuricemia, increasing the risk of cardiovascular and cerebrovascular diseases. In clinical practice, the diagnostic ability of PSIS should be improved, and pituitary function and complications should be evaluated in a timely manner to avoid delayed treatment.

## 1. Introduction

Pituitary stalk interruption syndrome (PSIS) is a rare disease initially reported in 1987, with a relatively low incidence rate of approximately 0.5 per 100,000 individuals [1–3]. It exhibits a higher prevalence among males compared to females [2]. Radiologically, PSIS manifests a classic triad: a thin or absent pituitary stalk, ectopic posterior pituitary, and anterior pituitary hypoplasia [1–3]. The interrupted stalk impedes hormones produced by the hypothalamus from reaching the pituitary, leading to varying levels of anterior pituitary hormone deficiency in patients with PSIS, and a smaller proportion may also experience impairment of posterior pituitary hormones [3]. Clinical manifestations vary based on the extent of pituitary stalk damage, primarily determined by the type and degree of hormone deficiency. Symptoms may include growth retardation, hypogonadotropic hypogonadism, hypoglycemia, hyponatremia, and central hypothyroidism [4, 5]. Most patients seek medical attention for growth retardation and hypogonadotropic hypogonadism, but some of them do not exhibit short stature [4, 6–8]. PSIS is prone to complications such as dyslipidemia, nonalcoholic fatty liver disease (NAFLD), and hyperuricemia [2, 4, 7, 8], increasing the risk of cardiovascular and cerebrovascular diseases. Due to the low incidence of PSIS, there is a lack of awareness and understanding in clinical practice, elevating the risk of misdiagnosis or underdiagnosis. This study systematically and retrospectively analyzed the clinical data and diagnostic and therapeutic measures of 24 PSIS patients. The aim is to enhance clinical diagnostic capabilities for PSIS, assess pituitary function and associated complications timely, and thereby avoid treatment delays.

## 2. Materials and Methods

### 2.1. Study Subjects and Methods

A total of 24 patients with PSIS, admitted to the Endocrinology Department from October 2013 to October 2023, were included. Information encompassed clinical presentation, laboratory data, imaging examinations, and management. The inclusion criteria were based on clinical presentation, laboratory data, and imaging examination confirming the diagnosis of PSIS. Exclusion criteria involved patients with chromosomal abnormalities or chronic systemic diseases. This systematic retrospective study was conducted at the First Affiliated Hospital of Sun Yat-sen University and received approval from the Ethics Committee of the hospital. All procedures adhered to the principles of the Helsinki Declaration. Informed consent for publication was obtained from each patient included in the study.

### 2.2. Laboratory Tests

#### 2.2.1. Hypothalamus-Pituitary-Growth Hormone Axis

The chemiluminescence assay was used to measure insulin-like growth factor-1 (IGF-1) and growth hormone. A growth hormone stimulation test (insulin-induced hypoglycemia test) was conducted, where a growth hormone peak value <5 ng/mL indicated complete growth hormone deficiency, and a growth hormone level in the range of 5−10 ng/mL indicated partial growth hormone deficiency.

#### 2.2.2. Hypothalamus-Pituitary-Gonadal Axis

The chemiluminescence assay was used to measure serum follicle stimulating hormone (FSH), luteinizing hormone (LH), prolactin (PRL), estradiol, progesterone, and testosterone. Gonadotropin-releasing hormone (GnRH) stimulation tests and human chorionic gonadotropin (HCG) stimulation tests were performed (sexual gland assessment was not performed in prepubertal individuals). For males, testosterone <1.58 ng/mL (normal range: 1.58–8.77 ng/mL), and for females, estradiol<27 pg/mL (follicular phase normal range: 27–122 pg/mL) with LH and FSH values normal or below normal, or during the GnRH stimulation test (intramuscular injection of triptorelin 100 *μ*g, LH levels measured at 0 min and 60 min), males with 60 min LH ≤4 IU/L and females with 60 min LH ≤6 IU/L were considered indicative of hypothalamus-pituitary-gonadal axis hypofunction [9]. In the HCG stimulation test, testosterone levels <3 ng/mL were considered indicative of testicular hypofunction [9].

#### 2.2.3. Hypothalamus-Pituitary-Adrenal Axis

The chemiluminescence assay was used to measure adrenocorticotropic hormone (ACTH), 8:00 am serum cortisol, and 24-h urinary free cortisol (UFC). 8:00 am serum cortisol <2.9 ug/dL (normal range: 2.9–19.4 ug/dL) and/or 24-h UFC <0.8 ug/dL (normal range: 0.8–11.7 *μ*g/dL) with ACTH levels normal or below normal range were defined as indicative of hypothalamic-pituitary-adrenal axis hypofunction.

#### 2.2.4. Hypothalamus-Pituitary-Thyroid Axis

The chemiluminescence assay was used to measure serum free triiodothyronine (FT3), free thyroxine (FT4), thyroid stimulating hormone (TSH), thyroid peroxidase antibody (TPOAb), and thyroid globulin antibody (TgAb). Hypothyroidism was defined as FT4 <7.5 pmol/L (normal range: 7.5–21.1 pmol/L).

#### 2.2.5. Biochemical Tests

The Beckman AU5800 fully automated biochemical analyzer from the United States was used to detect alanine aminotransferase (ALT), aspartate aminotransferase (AST), alkaline phosphatase (ALP), albumin, total bilirubin, total cholesterol (TC), triglycerides (TG), low-density lipoprotein-cholesterol (LDL-C), high-density lipoprotein-cholesterol (HDL-C), fasting plasma glucose (FPG), creatinine, urea nitrogen, uric acid, and electrolytes.

### 2.3. Imaging Examination

Three-millimeter contiguous sagittal and coronal plain T1-weighted, T2-weighted, and dynamic postcontrast T1-weighted magnetic resonance imaging (MRI) images through the pituitary were obtained using the Siemens Magnetom Skyra 3.0T MRI instrument from Germany. A left wrist X-ray was performed for bone age assessment, and an abdominal color Doppler ultrasound examination was performed for liver.

### 2.4. Statistical Analysis

Data analysis was conducted using SPSS 26.0 statistical software. Normal distribution of data was presented as mean ± standard deviation (SD), nonnormal distribution of data was presented as median (interquartile range), and categorical data were expressed by the numbers and percentages (*n*, %). A *p* value <0.05 was considered as statistically significant.

## 3. Result

### 3.1. Clinical Presentation

Twenty-four patients with PSIS were all confirmed by pituitary MRI and laboratory tests, including twenty-two males (91.7%) and two females (8.3%). The average age at diagnosis was 16 (11.5, 18.5) years. The average BMI was (21.21 ± 4.94) kg/m^2^, waist circumference was (77.94 ± 11.8) cm, waist-to-hip ratio was (0.88 ± 0.06), systolic blood pressure was (104.45 ± 15.47) mmHg, and diastolic blood pressure was (65.29 ± 7.49) mmHg (see Table 1 for details). Eight cases (33.3%) had a history of dystocia at birth, three cases (12.5%) had a breech or abnormal fetal position, one case (4.2%) had a history of resuscitation in the neonatal period, and one case (4.2%) had a childhood head injury. Fourteen cases (58.3%) were classified as short stature (height more than 2 standard deviations below the normal average for the same age and sex), including thirteen males with an average height of (152.57 ± 8.62) cm and one female with a height of 126 cm. Ten cases (41.7%) had normal height, including nine males with an average height of (171.22 ± 4.49) cm and one female with a height of 172 cm. Among the twenty-four patients, twelve had a history of growth hormone therapy, with six irregular users and six regular users. The duration of growth hormone therapy ranged from 0.2 to 2 years, and regular users showed a height increase of 10–30 cm. There was no blood relationship or relevant family history among all patients.

### 3.2. Endocrine Features

All twenty-four patients exhibited abnormalities in the secretion of the hypothalamic-pituitary axis hormone. Growth hormone deficiency was present in all twenty-four cases (100%), hypogonadism in twenty-four cases (100%), secondary adrenal insufficiency in twenty-two cases (91.7%), and hypothyroidism in twenty-one cases (87.5%). Among the patients, twenty cases (83.3%) showed abnormalities in four anterior pituitary hormones, three cases (12.5%) in three anterior pituitary hormones, and one case (4.2%) in two anterior pituitary hormones. The IGF-1 levels were low in all twenty-four patients. Based on age and gender-specific reference ranges, the IGF-1 assay results were converted to IGF-1 standard deviation scores (SDS) [10]. All twenty-four patients had IGF-1 SDS values <-2SDS. Insulin-induced hypoglycemia tests were conducted in all twenty-four patients, and none of them exhibited stimulated growth hormone secretion. Among the twenty-two male patients, none showed signs of pubertal development. Testicular volumes were measured using Prader orchidometer, with left testicular volumes ranging from 1 to 4 mL, an average volume of (1.65 ± 0.87) mL, and right testicular volumes ranging from 1 to 4 mL, an average volume of (1.65 ± 0.81) mL. Two cases presented cryptorchidism. The two female patients showed no pubic or axillary hair growth, no breast development, and no onset of menstruation. The hormone results of all twenty-four patients indicated hypogonadotropic hypogonadism, with sixteen patients undergoing a GnRH stimulation test, which showed a flat low curve with a weak response. The average value of LH at 60 minutes was (1.11 ± 2.47) IU/L. Five male patients underwent HCG stimulation test, but testosterone peak values were not elicited, with average value of (0.48 ± 0.21) ng/mL. The average PRL level in the twenty-four patients was (13.68 ± 8.58) ng/mL. The average 8:00 am serum cortisol level was (2.44 ± 2.64) *μ*g/dL, the average ACTH level was (4.36 ± 2.29) pmol/L, and the average 24-h UFC was below 0.8 *μ*g/dL. The average FT4 level was (6.82 ± 2.18) pmol/L, and the average TSH level was (3.74 ± 1.71) pmol/L. Both TPOAb and TgAb were negative. None of the twenty-four patients exhibited symptoms of polydipsia or polyuria, and the average urine specific gravity was (1.015 ± 0.006) (see Table 2 for details).

### 3.3. Biochemical Features

Among the twenty-four patients, nineteen cases (79.2%) had dyslipidemia (defined as TC>5.2 mmol/L, and/or TG>1.7 mmol/L, and/or LDL-C>3.4 mmol/L) [11], and nine cases (37.5%) had hyperuricemia (defined as uric acid>420 *μ*mol/L). The average values for TG, TC, LDL-C, ALT, AST, ALP, and uric acid were (1.79 ± 0.98) mmol/L, (5.75 ± 1.19) mmol/L, (3.87 ± 0.97) mmol/L, (46.13 ± 37.3) U/L, (48.63 ± 35.38) U/L, (164.7 ± 41.21) U/L, and (427.45 ± 125.93) *μ*mol/L, respectively. All these values were higher than the upper limits of the normal range. Blood albumin, total bilirubin, creatinine, urea nitrogen, serum calcium, serum phosphorus, serum sodium, and serum potassium were all within normal ranges (see Table 3 for details).

### 3.4. Radiological Features

**Figure 1:**
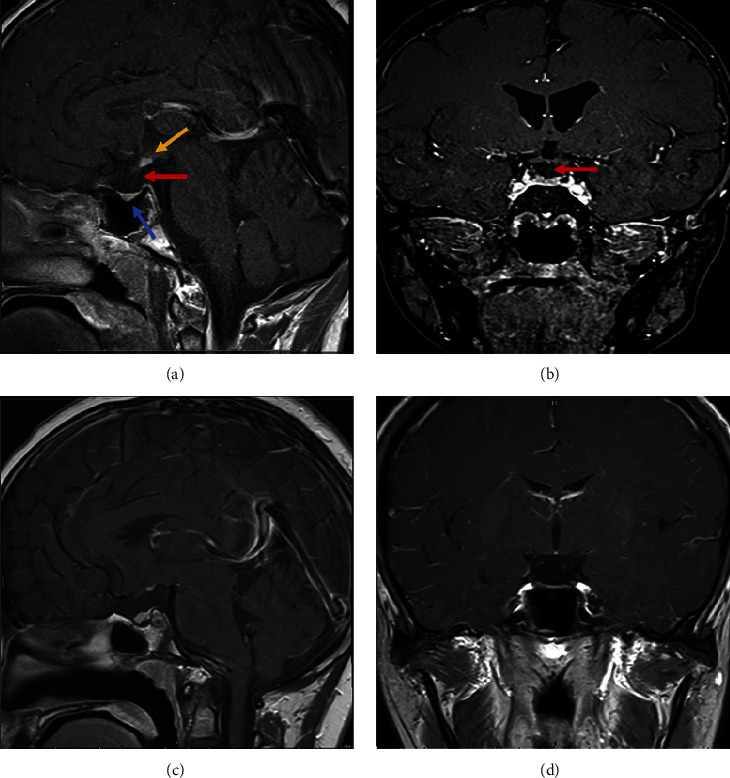
Pituitary magnetic resonance imaging (enhanced T1-weighted images). (a) The sagittal slice showed a thin pituitary stalk (red arrow), an ectopic posterior pituitary (yellow arrow), and anterior pituitary hypoplasia (blue arrow). (b) The coronal slice showed a thin pituitary stalk (red arrow). (c) The sagittal slice showed the absent pituitary stalk. (d) The coronal slice showed the absent pituitary stalk.

All twenty-four patients exhibit the typical PSIS triad of “a thin or absent pituitary stalk, ectopic posterior pituitary, and anterior pituitary hypoplasia” from a radiological perspective (Figure 1). Among them, thirteen cases showed the absence of the pituitary stalk, eleven cases presented with a thin pituitary stalk, and all twenty-four patients demonstrated an ectopic posterior pituitary and anterior pituitary hypoplasia. The average pituitary height of the twenty-four patients was (2.75 ± 1.25) mm. All the imaging diagnoses were performed by two experienced radiologists independently.

### 3.5. Imaging Examination

Bone age assessments for all twenty-four patients indicated delayed bone development. Fifteen cases (62.5%) were diagnosed with NAFLD based on ultrasound findings of hepatic steatosis, with or without liver enzyme abnormalities, and the exclusion of alcoholic liver disease, viral liver disease, autoimmune liver disease, drug-induced liver disease, and genetic metabolic liver disease [12].

### 3.6. Among the Twenty-Four PSIS Patients, Twelve Had No History of Growth Hormone Therapy

Among these twelve patients, four (33%) did not exhibit short stature. Specifically, three males had heights of 164 cm, 170 cm, and 174 cm, respectively, and one female had a height of 172 cm. Of these four patients, two had a history of dystocia at birth, one had a history of resuscitation in the neonatal period, and one had no special situation at birth. All four patients had growth hormone deficiency, hypogonadotropic hypogonadism, and secondary adrenal insufficiency. Three of these patients had hypothyroidism. Among the four patients, two exhibited PRL levels above the upper limit of the normal range, while the remaining two had PRL levels approaching the upper limit. The average PRL level for these four patients was (19.30 ± 2.12) ng/mL, which was significantly higher than the average PRL level of (12.55 ± 8.96) ng/mL observed in the other twenty patients. Furthermore, among these four patients, three had dyslipidemia, two had NAFLD, and two had hyperuricemia. Detailed clinical data for these four patients are presented in Table 4.

## 4. Treatment and Follow-Up

All 24 patients exhibited growth hormone deficiency. Among them, 10 patients (9 males and 1 female) with normal height at admission did not receive growth hormone supplementation. For the 14 patients with short stature, only 6 agreed to receive growth hormone therapy due to economic reasons. During the 1-2 years of follow-up, those who received growth hormone therapy showed varying degrees of height increase (5−10 cm/year). All 24 patients exhibited hypogonadism. For those with height requirements and unclosed epiphyses, growth hormone should be administered first. When the height reaches the desired level, sex hormones can be added. For patients without height requirements, supplementary sex hormones were administered directly. In males, testosterone was supplemented for androgen replacement, and combined treatment with human chorionic gonadotropin (HCG) and human menopausal gonadotropin (HMG) was used for spermatogenesis [9]. In females, estrogen and progestin were used to induce artificial menstrual cycles. Patients who did not adhere to a regular medication schedule showed no significant improvement in gonadal function. In contrast, those who followed a regular medication schedule demonstrated increased penile length and testicular volume in males, with the secretion of semen. Females experienced the resumption of menstrual cycles, along with an increase in the size of the uterus and ovaries. In 22 cases of secondary adrenal insufficiency, physiological doses of glucocorticoids were administered. None exhibited symptoms of adrenal insufficiency such as fatigue, nausea, or vomiting, and serum sodium, blood sugar, and blood pressure were all within the normal range. In 21 cases of hypothyroidism, supplementation of thyroid hormone was provided. Follow-up examinations showed normal thyroid function. In 19 cases of dyslipidemia, statins and/or fenofibrate were administered, along with lifestyle interventions. Follow-up examinations showed normal blood lipid levels. And patients who received growth hormone therapy, sex hormone supplementation, or thyroid hormone replacement had a faster time to attain normal blood lipid levels. In 15 cases of NAFLD, 10 cases had abnormal liver enzymes. Treatment involved the administration of polyene phosphatidylcholine capsules and/or diammonium glycyrrhizinate enteric-coated capsules for liver protection. Follow-up examinations showed normal liver function. In 9 cases of hyperuricemia, treatment included benzbromarone for uric acid reduction and lifestyle interventions. Follow-up examinations showed normal uric acid levels.

## 5. Discussion

PSIS is a rare clinical disease characterized by the characteristic triad of “a thin or absent pituitary stalk, ectopic posterior pituitary, and anterior pituitary hypoplasia.” In this study, all patients were found to have the classic imaging features. Due to thinning or absence of pituitary stalks, hormones secreted by the hypothalamus cannot be transported to the pituitary, leading to clinical symptoms. Most patients present with growth retardation and hypogonadotropic hypogonadism. The age at diagnosis in our study was 16 years old, which is older than what has been reported previously [1]. All 24 patients exhibited growth hormone deficiency, as evidenced by the insulin-induced hypoglycemia test. None of the patients had pubertal development, and results of sex hormone assessments indicated hypogonadotropic hypogonadism. Secondary adrenal insufficiency was observed in 22 cases (91.2%), but none experienced an adrenal crisis. Hypothyroidism was observed in 21 cases (87.5%), but none experienced a thyroid crisis. None of the 24 cases experienced diabetes insipidus. The types and degrees of pituitary hormone deficiencies in this study were similar to those reported in previous studies [6]. Zhang et al. reported a case of PSIS in which, despite discontinuation of glucocorticoid and thyroid hormone replacement therapy, the patient never experienced an adrenal or thyroid crisis [4]. This could be attributed to the maintenance of metabolism in a nonstress state by low levels of ACTH, cortisol, and thyroid hormones in the patient [4]. Ma and Ning reported a case of PSIS combined with acute leukemia, where an adrenal crisis occurred, likely associated with leukemia and infection-induced stress [13]. In the absence of stressors, PSIS is less likely to manifest as an adrenal or thyroid crisis [1, 13]. The occurrence of diabetes insipidus is uncommon in PSIS [3]. This phenomenon may be related to the formation of ectopic posterior pituitary [3], and the mechanism is still unclear.

In this study, 12 patients had a history of growth hormone therapy before admission, while 12 have not received the treatment. Among the 12 patients without a history of growth hormone therapy, 4 cases (33%) did not exhibit short stature. Specifically, three males had heights of 164 cm, 170 cm, and 174 cm, respectively, and one female had a height of 172 cm. Although the bone age of all patients was delayed and growth hormone levels were low, 4 patients had normal height without a history of growth hormone therapy. This phenomenon in PSIS has been previously reported by several studies [3, 4, 6–8]. Some researchers suggest that PSIS with normal height is more common in patients with deficiencies in multiple pituitary hormones, especially gonadotropin deficiency [6, 8]. Insufficient sex hormones can delay epiphyseal closure, resulting in a normal or taller final height even though these patients have a severe deficiency in growth hormone [6, 8]. The 4 patients in this study all presented with hypogonadotropic hypogonadism and delayed bone age development, yet they attained normal height despite growth hormone deficiency, suggesting the possibility of sex hormone insufficiency leading to height growth. Additionally, previous studies have reported a phenomenon that normal growth without growth hormone might be associated with hyperinsulinemia, hyperprolactinemia, elevated leptin levels, and growth hormone variants [4, 8]. Hyperinsulinemia or hyperprolactinemia can alter the distribution of circulating IGF-1, which can promote growth even in the absence of growth hormone [4, 8]. The leptin secreted by adipose tissue induced by obesity may serve as a bone growth factor for patients with growth hormone deficiency, playing a role in maintaining growth velocity [8]. In this study, 4 patients without a history of growth hormone treatment with no short stature were not obese, and their average PRL level was (19.30 ± 2.12) ng/mL, significantly higher than the average PRL level of (12.55 ± 8.96) ng/mL observed in the other 20 patients. Therefore, it cannot be ruled out that the increase in height is related to a relatively high level of prolactin. Furthermore, this study observed one patient whose height increased from 145 cm at 20 years old to 174 cm at 27 years old without growth hormone treatment, showing a growth of 29 cm in 7 years. Similar phenomena have been reported by Wang et al., where a patient increased in height by 30 cm after the age of 25 without growth hormone treatment, reaching a normal height [7]. This suggests that patients with PSIS exhibit a phenomenon of lagging catch-up growth [7]. The reason why PSIS patients with growth hormone deficiency can achieve a normal height without growth hormone treatment remains unclear. However, this phenomenon indicates that clinicians should be vigilant for PSIS in patients with normal height but gonadal developmental abnormalities and promptly conduct pituitary function and imaging examinations to confirm the diagnosis.

PSIS leads to various hormone deficiencies in the pituitary, affecting not only growth and gonadal development but also influencing human metabolism. This study observed a high incidence of dyslipidemia (79.2%), NAFLD (62.5%), and hyperuricemia (37.5%) in patients with PSIS, all of which are risk factors for cardiovascular and cerebrovascular diseases. Without timely screening and detection, these conditions may exacerbate the progression of the diseases. Growth hormone plays a role in promoting triglyceride breakdown in adipose tissue, regulating the activity of lipoprotein lipase and the quantity of LDL-C receptors [14]. In the absence of growth hormone, levels of LDL-C and triglycerides tend to increase [14]. A retrospective cohort study reported a negative correlation between IGF-1 levels and LDL-C in patients with growth hormone deficiency [15]. Insufficient sex hormones can also lead to dyslipidemia. In cases of androgen deficiency, there is a decrease in lean body mass and an increase in fat mass, resulting in dyslipidemia [16]. In addition, animal experiments have confirmed that TC and LDL-C levels significantly increase in ovariectomized mice [17]. Su et al. reported that thyroid hormone plays an important role in lipid metabolism [18]. Hypothyroidism is closely associated with increased serum levels of TC, LDL-C, and TG [18]. PSIS is prone to being associated with dyslipidemia, but prompt hormone replacement therapy can help improve dyslipidemia. Studies have suggested that growth hormone replacement therapy normalizes IGF-1, reduces TC and LDL-C, and improves lipid profile [15, 19]. Testosterone replacement therapy is also associated with lower serum concentrations of TC [20]. One meta-analysis reveals that testosterone supplementation in hypogonadal men reduces body fat and increases lean body mass, reducing the risk of coronary artery disease [21]. In addition, animal experiments have shown that estrogen replacement therapy lowers lipids by increasing serum estrogen levels and estrogen receptor expression in ovariectomized mice [17]. Dong et al. reported that there is a significant decrease in serum levels of TC, LDL-C, and TG after thyroid hormone replacement therapy in patients with hypothyroidism [22]. Therefore, timely hormone replacement in PSIS can help improve lipid metabolism and reduce the risk of cardiovascular and cerebrovascular diseases.

In addition to dyslipidemia, PSIS is closely associated with NAFLD [2, 23]. NAFLD encompasses a spectrum of liver diseases, progressing from nonalcoholic fatty liver (NAFL) and nonalcoholic steatohepatitis (NASH) to cirrhosis [12, 23]. The severity of growth hormone deficiency is positively correlated with the severity of hepatic steatosis in NAFLD [2]. Previous study has reported that growth hormone deficiency inhibits the activation of signal transducer and activator of transcription-5 (STAT-5), increases liver lipid uptake, and promotes phosphorylation of STAT-1 and STAT-3. The activation of STAT-1 and STAT-3 has been shown to promote the development of NAFLD [23]. Moreover, insufficient sex hormones also increase the risk of NAFLD. Sarkar et al. reported an increased incidence of NASH with decreasing testosterone levels [24]. Testosterone replacement therapy has been shown to reduce intrahepatic triglyceride content and improve hepatic steatosis [25, 26]. In the case of estrogen deficiency, there is a dysregulation of hepatic lipid metabolism, promoting the formation of fatty liver [27]. Estrogen replacement therapy can reduce hepatic fat deposition [2]. Hypothyroidism also plays a significant role in the development of NAFLD. Both a meta-analysis and a retrospective case analysis have reported a significant association between hypothyroidism and NAFLD [28, 29]. Some researchers have estimated the time for progression from NAFLD to cirrhosis to be around 57 years [30]. However, in patients with pituitary dysfunction, the average time for NAFLD to progress from abnormal liver function to decompensated cirrhosis is reported to be 6.9 years [31], progressing rapidly. Therefore, liver changes in patients with PSIS are of concern.

This study also observed that PSIS is prone to be accompanied by hyperuricemia. Mandal et al. reported that IGF-1 can reduce uric acid levels by activating uric acid secretion transporters and inhibiting insulin [32]. When there are insufficient IGF-1 levels, uric acid levels tend to increase. Sesti et al. indicate a negative correlation between IGF-1 and uric acid levels in nondiabetic adults [33]. Insufficiency in sex hormones is also associated with hyperuricemia [34]. Estrogen increases uric acid excretion by influencing uric acid reabsorption-related transporters and reduces uric acid production by maintaining lipid metabolism stability [35]. When estrogen is insufficient, uric acid rises. Additionally, when there is a deficiency in thyroid hormones, the reduction in myocardial contractility and cardiac output leads to decreased renal blood flow and glomerular filtration rate, resulting in reduced uric acid excretion and elevated uric acid levels [36]. Some studies have reported a significant reduction in uric acid levels in patients with hypothyroidism after thyroidectomy when undergoing L-T4 replacement therapy [36]. PSIS is prone to complications such as dyslipidemia, NAFLD, and hyperuricemia. Therefore, in addition to early identification of PSIS, clinicians should promptly assess lipid profiles, uric acid levels, and liver conditions. Timely screening for complications can help reduce the occurrence of cardiovascular and cerebrovascular events.

The pathogenesis of PSIS is still unclear, with perinatal injury and genetic factors being the two major causes. Some scholars suggest that perinatal injuries such as dystocia, breech delivery, and neonatal hypoxemia may contribute to the development of PSIS [2, 37], while others suggest an association between PSIS and genetic factors. These genetic factors include mutations in PROP1, HESX1, LHX3/LHX4, and OTX2 genes, as well as mutations in Wnt, Notch, and Shh signaling pathways [3, 4, 38]. After the diagnosis of PSIS, timely hormone replacement therapy should be initiated, following the principle of replenishing what is deficient. In cases where there is concurrent secondary adrenal insufficiency and hypothyroidism, glucocorticoid supplementation should precede thyroid hormone replacement. In the presence of growth hormone deficiency and hypogonadism, treatment should be combined with height requirements. If height is suboptimal and the epiphyseal plates are not closed, growth hormone should be administered first. When the height reaches the desired level, sex hormones can be added. If the height is already at the desired level or the epiphyseal plates are closed, sex hormone replacement should be initiated immediately. When PSIS induces changes in pituitary hormones levels, it can also lead to metabolic alterations in the human body, resulting in abnormalities such as dyslipidemia, NAFLD, and hyperuricemia. Timely intervention with lipid-lowering, liver protection, and uric acid lowering measures should be implemented to reduce the risk of cardiovascular and cerebrovascular diseases.

In this study, we have some limitations. Firstly, we lack genetic analysis of 24 patients, which prevents the analysis of the characteristics of PSIS from a genetic perspective. For some patients without short stature, it remains to be explored whether genetic screening can predict the height of PSIS patients, which could potentially save the economic cost of growth hormone therapy for patients. Additionally, our study observed 24 PSIS. Among them, 13 cases showed the absence of the pituitary stalk, while 11 cases presented with a thin pituitary stalk. The occurrence of pituitary stalk absence was higher than that of pituitary stalk thinning, which is consistent with previous study [6]. However, due to the small sample size, no comparative analysis was conducted between the two groups. It remains unclear whether the visibility of the pituitary stalk on MRI can predict the severity of hormone deficiencies in PSIS. Further study is needed to help better understand PSIS.

## 6. Conclusion

This study systematically and retrospectively analyzed clinical data from 24 patients with PSIS. The patients were initially presented late, often seeking medical attention due to growth retardation and abnormal gonadal development. Some patients did not exhibit short stature. PSIS is prone to be associated with dyslipidemia, NAFLD, and hyperuricemia. In clinical practice, it is important to enhance the diagnostic ability for PSIS, timely assess pituitary function and complications, achieve early detection, early diagnosis, and early treatment, and avoid delaying the optimal treatment time.

## Figures and Tables

**Table 1:** Clinical presentation in 24 PSIS patients.

Characteristics	Results
Age at diagnosis (year)	16 (11.5, 18.5)
Gender (male/female) %	91.7%/8.3%
BMI (kg/m^2^)	21.21 ± 4.94
Waist circumference (cm)	77.94 ± 11.8
WHR	0.88 ± 0.06
SBP (mmHg)	104.45 ± 15.47
DBP (mmHg)	65.29 ± 7.49
Birth history	
Dystocia, *n* (%)	8 (33.3%)
Abnormal fetal position, *n* (%)	3 (12.5%)
Resuscitation in neonatal period, *n* (%)	1 (4.2%)
Head injury, *n* (%)	1 (4.2%)
No special situation, *n* (%)	11 (45.8%)
Short stature, *n* (%)	14 (58.3%)
History of growth hormone therapy before admission, *n* (%)	12 (50%)

Data are expressed as mean ± standard deviation, median (interquartile range), and numbers (percentage). BMI, body mass index; WHR, waist to hip ratio; SBP, systolic blood pressure; DBP, diastolic blood pressure.

**Table 2:** Endocrine features in 24 PSIS patients.

Parameters	Results	Normal range
IGF-1<-2 SDS, *n* (%)	24 (100%)	
Growth hormone stimulation test: growth hormone peak value <5 ng/mL, *n* (%)	24 (100%)	
Growth hormone deficiency, *n* (%)	24 (100%)	
Testosterone (ng/mL)	<0.13^*∗*^	Male 1.58–8.77
		Female (follicular phase) 0.13–1.30
Estradiol (pg/mL)	<10^*∗*^	Male<44
		Female (follicular phase) 27–122
FSH (IU/L)	0.84 ± 1.14	Male 1–8
		Female (follicular phase) 3.85–8.78
LH (IU/L)	0.31 ± 0.81	Male 2–12
		Female (follicular phase) 2.12-10.89
PRL (ng/mL)	13.68 ± 8.58	Male 1.61-18.77, female 3.34-26.72
Hypogonadism, *n* (%)	24 (100%)	
8:00 am serum cortisol (*μ*g/dL)	2.44 ± 2.64	2.9–19.4
ACTH (pmol/L)	4.36 ± 2.29	0-10.2
24-h UFC (*μ*g/dL)	<0.8^*∗*^	0.8–11.7
Hypoadrenocorticism, *n* (%)	22 (91.2%)	
FT3 (pmol/L)	4.34 ± 0.85	2.63–5.7
FT4 (pmol/L)	6.82 ± 2.18	7.5–21
TSH (mIU/L)	3.74 ± 1.71	0.3–5.95
Hypothyroidism, *n* (%)	21 (87.5%)	
Urine specific gravity	1.015 ± 0.006	1.005–1.030
Diabetes insipidus, *n* (%)	0 (0%)	
With 4 hormone deficiencies, *n* (%)	20 (83.3%)	
With 3 hormone deficiencies, *n* (%)	3 (12.5%)	
With 2 hormone deficiencies, *n* (%)	1 (4.2%)	

^*∗*^Below detection value. Data are expressed as mean ± standard deviation and numbers (percentage). IGF-1, insulin-like growth factor-1; SDS: standard deviation score; FSH, follicle stimulating hormone; LH, luteinizing hormone; PRL, prolactin; ACTH, adrenocorticotropic hormone; 24-h UFC, 24-h urinary free cortisol; FT3, free triiodothyronine; FT4, free thyroxine; TSH, thyroid stimulating hormone.

**Table 3:** Biochemical features in 24 PSIS patients.

Parameters	Results	Normal range
ALT (U/L)	46.13 ± 37.3	1–40
AST (U/L)	48.63 ± 35.38	1–37
ALP (U/L)	164.7 ± 41.21	0–110
Blood albumin (g/L)	45.8 ± 2.52	35–50
Total bilirubin (*μ*mol/L)	12.78 ± 4.84	3–22
TC (mmol/L)	5.75 ± 1.19	3.1–5.2
TG (mmol/L)	1.79 ± 0.98	0.33–1.7
LDL-C (mmol/L)	3.87 ± 0.97	1.94–3.40
HDL-C (mmol/L)	1.09 ± 0.28	1.09–1.63
FBG (mmol/L)	5.05 ± 1.15	2.9–6.0
Creatinine (umol/L)	60.79 ± 12.85	53–115
Urea nitrogen (mmol/L)	4.54 ± 1.29	2.9–8.6
Uric acid (*μ*mol/L)	427.45 ± 125.93	200–420
Serum calcium (mmol/L)	2.39 ± 0.11	2.1–2.6
Serum phosphorus (mmol/L)	1.45 ± 0.27	0.97–1.62
Serum sodium (mmol/L)	139.21 ± 2.23	135–145
Serum potassium (mmol/L)	4.05 ± 0.31	3.5–5.3
Dyslipidemia, *n* (%)	19 (79.2%)	
NAFLD, *n* (%)	15 (62.5%)	
Hyperuricemia, *n* (%)	9 (37.5%)	

Data are expressed as mean ± standard deviation, median (interquartile range), and numbers (percentage). ALT, alanine aminotransferase; AST, aspartate aminotransferase; ALP, alkaline phosphatase; TC, total cholesterol; TG, triglycerides; LDL-C, low-density lipoprotein-cholesterol; HDL-C, high-density lipoprotein-cholesterol; FPG, fasting plasma glucose; NAFLD, nonalcoholic fatty liver disease.

**Table 4:** Clinical characteristics in PSIS patients of normal height (with no history of growth hormone therapy).

Parameters	Patient 1	Patient 2	Patient 3	Patient 4	Normal range
Age at diagnosis (year)	27	11	30	15	

Gender	Male	Male	Male	Female	

Height (cm)	174	170	164	172	
BMI (kg/m^2^)	24.3	20	14.35	22.3	
Waist circumference (cm)	88	71	64	75	
WHR	0.9	0.77	0.85	0.8	
SBP (mmHg)	125	92	117	131	
DBP (mmHg)	66	61	78	67	

Birth history	Dystocia	No special situation	Resuscitation in neonatal period	Dystocia	

IGF-1(ng/mL)	<25^*∗*^	36.7	<25^*∗*^	58.02	
IGF-1<-2 SDS	Yes	Yes	Yes	Yes	
Testosterone (ng/mL)	<0.13^*∗*^	0.28	0.37	<0.13^*∗*^	Male 1.58–8.77
					Female (follicular phase) 0.13-1.30
Estradiol (pg/mL)	<10^*∗*^	<10^*∗*^	<10^*∗*^	<10^*∗*^	Male <44
					Female (follicular phase) 27–122
FSH (IU/L)	0.15	0.38	0.19	0.06	Male 1–8
					Female (follicular phase) 3.85–8.78
60 min FSH (IU/L)	0.51	Not detected	0.69	0.22	
LH (IU/L)	0.04	0.07	0.1	0.02	Male 2–12
					Female (follicular phase) 2.12-10.89
60 min LH (IU/L)	0.06	Not detected	0.25	0.04	
PRL (ng/mL)	19.75	22	16.99	18.46	Male 1.61-18.77
					Female 3.34-26.72
8:00 am serum cortisol (ug/dL)	<0.8∗	2.6	2.6	0.9	2.9–19.4
ACTH (pmol/L)	5.45	6.6	5.15	5.45	0-10.2
24-h UFC (*μ*g/dL)	<0.8^*∗*^	<0.8^*∗*^	<0.8^*∗*^	<0.8^*∗*^	0.8–11.7
FT3 (pmol/L)	4.8	4.0	4.43	4.6	2.63–5.7
FT4 (pmol/L)	4.6	11^*∗∗*^	7.56	4.4	7.5–21
TSH (mIU/L)	1.76	2.36	2.92	5.94	0.3–5.95
Number of axes with hormone deficiencies	4	4	3	4	
ALT (U/L)	26	15	14	36	1–40
AST (U/L)	34	27	30	37	1–37
TC (mmol/L)	6.7	5.5	4.6	5.4	3.1–5.2
TG (mmol/L)	1.93	0.6	0.64	2.4	0.33–1.7
LDL-C (mmol/L)	4.94	3.58	2.63	3.38	1.94–3.4
Uric acid (*μ*mol/L)	596	325	374	443	200–420
NAFLD	Yes	No	No	Yes	

^*∗*^Below detection value. ^*∗∗*^Treatment with levothyroxine. BMI, body mass index; WHR, waist to hip ratio; SBP, systolic blood pressure; DBP, diastolic blood pressure; IGF-1, insulin-like growth factor-1; SDS: standard deviation score; FSH, follicle stimulating hormone; 60 min FSH, FSH was measured at 60 minutes following intramuscular injection triptorelin 100 ug; LH, luteinizing hormone; 60 min LH, LH was measured at 60 minutes following intramuscular injection triptorelin 100 ug; PRL, prolactin; ACTH, adrenocorticotropic hormone; 24-h UFC, 24-h urinary free cortisol; FT3, free triiodothyronine; FT4, free thyroxine; TSH, thyroid stimulating hormone; ALT, alanine aminotransferase; AST, aspartate aminotransferase; TC, total cholesterol; TG, triglycerides; LDL-C, low-density lipoprotein-cholesterol; NAFLD, nonalcoholic fatty liver disease.

## Data Availability

The data used to support this study are available on request.
